# Bilateral Tubal Gestation Associated with Schistosomiasis in an African Woman

**DOI:** 10.1155/2014/674514

**Published:** 2014-12-14

**Authors:** K. H. Odubamowo, O. M. Akinpelu, O. O. Lawal, C. A. Okolo, A. A. Odukogbe, A. O. Adekunle

**Affiliations:** ^1^Department of Obstetrics and Gynaecology, University College Hospital, Ibadan 20004, Oyo State, Nigeria; ^2^Department of Pathology, University College Hospital, Ibadan 20004, Oyo State, Nigeria

## Abstract

*Background*. The incidence of tubal ectopic gestation caused by schistosomiasis induced tubal pathology is undocumented in this environment, which may be due to rarity of this pathology. Bilateral tubal gestation is common in patients that have undergone in vitro fertilization. We report a hitherto undocumented case of spontaneous bilateral ectopic gestation following tubal schistosomiasis. *Case Report*. Mrs. OB was a 32-year-old G4P3^+0^ (3 alive) woman who complained of abdominal pain and bleeding per vaginam of 4 and 2 days' duration respectively following 8 weeks of amenorrhea. A clinical impression of ruptured ectopic gestation was confirmed by ultrasound scanning. She had bilateral salpingectomy with histology of specimens showing bilateral ectopic gestation with *Schistosoma haematobium* induced salpingitis (findings of *Schistosoma haematobium* ova noted on slide). *Conclusion*. *Schistosoma* induced salpingitis is a rare but possible cause of bilateral tubal gestation.

## 1. Introduction

Bilateral ectopic gestation is a rare clinical condition, with an incidence of 1 per 200,000 pregnancies [1]. It is common in patients that underwent in vitro fertilization for assisted conception [2, 3] and it has also been reported in a woman using oral contraception [4]. There has been no reported case of bilateral tubal gestation following schistosomiasis, although a few cases of unilateral tubal gestation have been reported [5–7]. The pathophysiologic mechanism implicated in this is mechanical obstruction of the tubes by the schistosome ova lodged in the peritubal vasculature with associated fibrosis and distortion of the tubes [7, 8].

## 2. Case Report

Mrs. OB was a 32-year-old G4P3^+0^  (3 alive) trader who presented on the 5th of March, 2013, in the gynaecological emergency room with abdominal pain of 4 days' duration and bleeding per vaginam of 2 days' duration following 8 weeks of amenorrhea.

Examination revealed that she was pale and dehydrated with a respiratory rate of 28 excursions per minute, pulse rate was 104 beats per minute, and blood pressure was 134/74 mmHg. There was generalized abdominal tenderness; pelvic examination revealed cervical os that was closed, with tenderness in the fornices, and the pouch of Douglas was full. The uterine size could not be assessed because of tenderness.

A clinical impression of suspected ruptured tubal gestation was made; urine pregnancy test was positive. Packed cell volume was 18%. Urgent abdominopelvic ultrasound scanning revealed bulky uterus measuring 11.37 × 4.83 cm in its longitudinal and transverse diameters. It showed normal myometrial echogenicity. There was a decidual reaction within the endometrium. A large mixed echogenic structure with irregular and ill-defined margins was seen in the right adnexum suggestive of ruptured ectopic gestation with surrounding hematoma. There was gross increase in intraperitoneal fluid suggestive of haemoperitoneum.

The patient was resuscitated with intravenous fluids and informed consent for exploratory laparotomy was obtained. Intraoperative findings included 2.7 litres of haemoperitoneum, ruptured right tubal ampullary gestation, and bulbous fatty mass at the fimbrial portion of the left tube measuring 4 cm × 6 cm, and the uterus was bulky. At laparotomy she had bilateral salpingectomy.

The patient was transfused with 3 units of whole blood. The posttransfusion packed cell volume was 25%. She made significant improvement and she was discharged home on the 7th postoperative day.

She was seen in gynaecological clinic 2 weeks later with histology report of specimens which revealed left and right fallopian tubes showing distension of the lamina by chorionic villi of varying sizes, hemorrhage, and decidualized stroma. Also seen are multiple granulomas with calcified ova of* S. haematobium* with their characteristic terminal spines, features consistent with bilateral ectopic gestation with* Schistosoma* induced salpingitis. She was comanaged with a parasitologist. She, however, declined urine and stool microscopy before using oral praziquantel (20 mg/kg twice a day for one day), after which urine and stool microscopy for ova of parasites done a month after medication was negative.

## 3. Discussion

**Figure 1:**
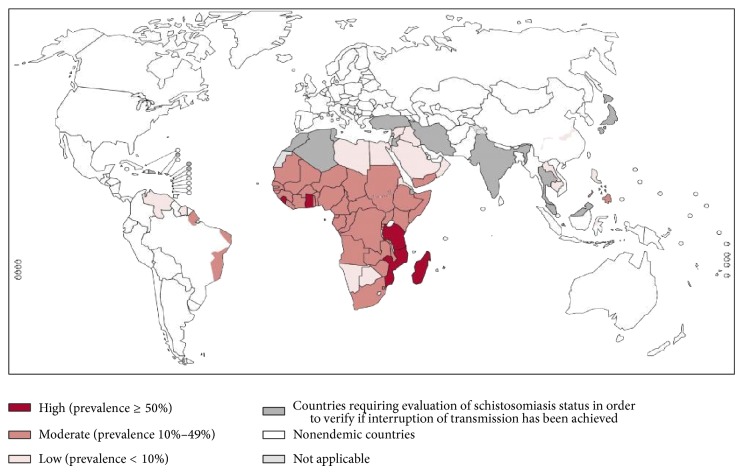
Distribution of schistosomiasis, worldwide, 2011 [9].

The case presented illustrates that schistosomiasis is a cause of tubal damage and ectopic gestation. The infestation is preventable and 85% of cases are found in sub-Saharan Africa [10] (Figure 1) with Nigeria being one of the 74 countries endemic for schistosomiasis. It is common in these tropical regions because of the poor living conditions with reliance on streams and other water bodies for their daily chores and other activities like farming and fishing [11–13]. Although the case presented occurred in an endemic area, female genital schistosomiasis may occur as well in returning travellers with history of contact with fresh water bodies in endemic countries [11, 13].

The most common specie is* Schistosoma (S.) haematobium* which infests the urinary bladder with resultant hematuria.* S. haematobium* resides in the pelvic and vesical plexus while* Schistosoma mansoni* is found in the portal venous system, which makes lesions caused by them to be common in the bladder and the rectum, respectively [6, 7, 10].

Infection is contracted by contact with fresh water containing the cercariae larva (released from the water snail, the intermediate host) which penetrates the human skin. The water snails are infected by the miracidia which develop from the ova excreted from infected humans. Infected snails reinfect humans to continue the cycles [11, 13].

Female genital schistosomiasis (FGS) is the presence of sandy patches and/or ova of parasites in genital tissue [14]. The diagnosis is made if there is a chronic form of genital schistosomiasis with no other symptoms [11].* S. haematobium* is the commonest cause of FGS occurring in 50–80% of parasitized females [15]. Histopathologic examination of surgical specimens has shown that* S. haematobium* ova can be found in all female genital organs but most commonly the cervix and vagina but rarely tubal specimens [12, 16–18]. The incidence of tubal pregnancy is about 60% with tubal schistosomiasis compared to 33% in patients without it [7]. The pathophysiologic mechanism is said to be due to granulomatous reaction leading to mechanical obstruction or tubal fibrosis caused by ischemia of the tubes after occlusion of the blood vessels by the eggs of the parasite [6, 7].

**Figure 2:**
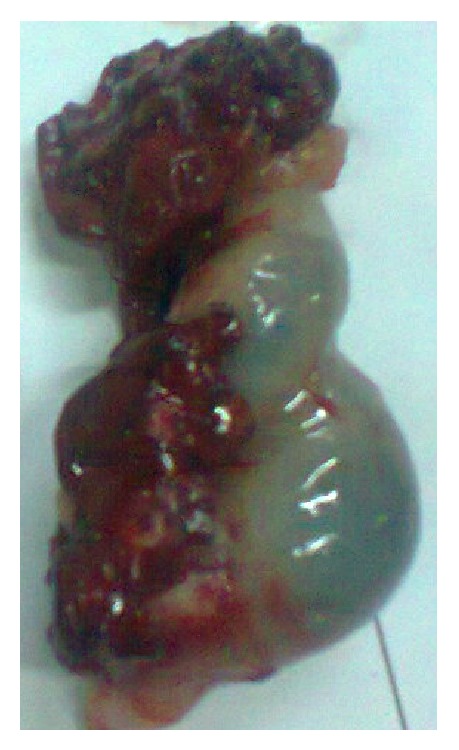
Ruptured right fallopian tube.

**Figure 3:**
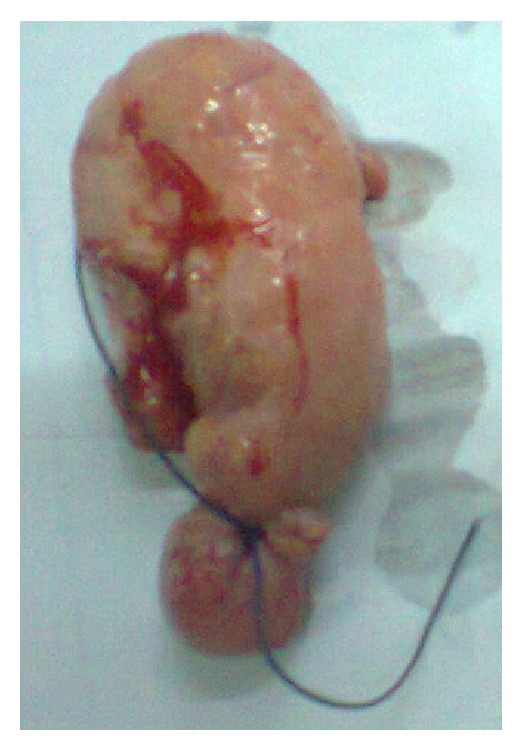
Bulbous left fallopian tube.

**Figure 4:**
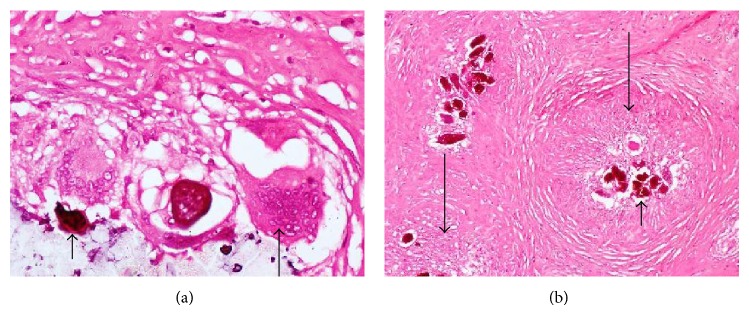
Photomicrographs showing calcified schistosome ova (short arrows), inflammatory giant cell (medium arrow), and granuloma surrounding the ova (long arrows), in the right tube.

**Figure 5:**
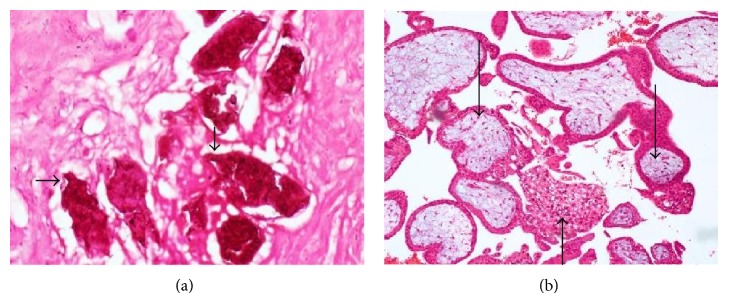
Photomicrographs showing* S. haematobium* ova with characteristic terminal spines (short arrows), granuloma (medium arrow), and chorionic villi in the left tube (long arrows).

This was a case of bilateral tubal pregnancy with rupture of the right tube (Figure 2) which resulted in haemoperitoneum; the left tube (Figure 3) was bulbous with fatty appearance due to granulomatous reaction induced by the infestation. Granulomas can be seen in Figure 4 (long arrows), while* S. haematobium* ova with their characteristic terminal spines can be seen in Figure 5 (short arrows).

This case shows that schistosomiasis is a rare but possible cause of bilateral ectopic gestation; it also illustrates the importance of careful examination of the second tube during laparotomy for an ectopic gestation. It highlights the importance of vector control in the prevention of ectopic gestation which is the commonest cause of mortality from gynaecological emergencies.
